# SARS-CoV-2 Disease through Viral Genomic and Receptor Implications: An Overview of Diagnostic and Immunology Breakthroughs

**DOI:** 10.3390/microorganisms9040793

**Published:** 2021-04-10

**Authors:** Alessio Danilo Inchingolo, Angelo Michele Inchingolo, Ioana Roxana Bordea, Giuseppina Malcangi, Edit Xhajanka, Antonio Scarano, Felice Lorusso, Marco Farronato, Gianluca Martino Tartaglia, Ciro Gargiulo Isacco, Grazia Marinelli, Maria Teresa D’Oria, Denisa Hazballa, Luigi Santacroce, Andrea Ballini, Maria Contaldo, Francesco Inchingolo, Gianna Dipalma

**Affiliations:** 1 Department of Interdisciplinary Medicine, University of Medicine Aldo Moro, 70124 Bari, Italy; ad.inchingolo@libero.it (A.D.I.); angeloinchingolo@gmail.com (A.M.I.); drciroisacco@gmail.com (C.G.I.); graziamarinelli@live.it (G.M.); mtdoria51@gmail.com (M.T.D.); denisahazballa@gmail.com (D.H.); luigi.santacroce@uniba.it (L.S.); francesco.inchingolo@uniba.it (F.I.); giannadipalma@tiscali.it (G.D.); 2 Department of Oral Rehabilitation, Faculty of Dentistry, Iuliu Hațieganu University of Medicine and Pharmacy, 400012 Cluj-Napoca, Romania; 3 Department of Dental Prosthesis, Medical University of Tirana, Rruga e Dibrës, U.M.T., 1001 Tirana, Albania; editxhajanka@yahoo.com; 4 Department of Innovative Technologies in Medicine and Dentistry, University of Chieti-Pescara, 66100 Chieti, Italy; a.scarano@unich.it; 5 UOC Maxillo-Facial Surgery and Dentistry, Department of Biomedical, Surgical and Dental Sciences, School of Dentistry, Fondazione IRCCS Ca Granda, Ospedale Maggiore Policlinico, University of Milan, 20100 Milan, Italy; marco.farronato@unimi.it (M.F.); gianluca.tartaglia@unimi.it (G.M.T.); 6 Director of Research at Human Stem Cells Research Center HSC, Ho Chi Minh 70000, Vietnam; 7 Embryology and Regenerative Medicine and Immunology at Pham Chau Trinh University of Medicine, Hoi An 51300, Vietnam; 8 Department of Medical and Biological Sciences, University of Udine, Via delle Scienze, 206, 33100 Udine, Italy; 9 Kongresi Elbasanit, Rruga: Aqif Pasha, 3001 Elbasan, Albania; 10 Department of Biosciences, Biotechnologies and Biopharmaceutics, Campus Universitario Ernesto Quagliariello, University of Bari “Aldo Moro”, 70125 Bari, Italy; andrea.ballini@uniba.it; 11 Department of Precision Medicine, University of Campania Luigi Vanvitelli, 80138 Naples, Italy; 12 Multidisciplinary Department of Medical-Surgical and Dental Specialties, University of Campania Luigi Vanvitelli, Via Luigi de Crecchio, 6, 80138 Naples, Italy; maria.contaldo@unicampania.it

**Keywords:** SARS-CoV-2 (COVID-19 pandemic), ACE2, TMPRSS2, oral mucosa (salivary glands), furin, Cytokine Storm Syndrome (CSS), microbiome, vaccines

## Abstract

The SARS-CoV-2 (severe acute respiratory syndrome coronavirus 2), which is believed to have originated in China towards the end of November 2019, has now spread across the globe, causing a pandemic in 192 countries. The World Health Organization has called it the SARS-CoV-2 pandemic. Rapid dissemination of the virus occurs mainly through the saliva (Flügge’s droplets) and aerosol, together with nasal and lachrymal passages. The literature associated with the recent advancement in terms of rapid diagnostics and SARS-CoV-2 vaccines has thoroughly studied the role of ACE2 receptors and Furin, as well as viral agent access into the host cell and its significant persistence at the level of the oral mucosa, which represents the main access to the virus. The purpose of this review was to underline the processes of SARS-CoV-2 infection mechanisms and novel breakthroughs in diagnostics and vaccines. Different technologies, such as the RT-PCR molecular test and the antigenic test, have been developed to identify subjects affected by the SARS-CoV-2 in order to improve the tracking of infection geographical diffusion. Novel rapid and highly sensitive diagnostic tests has been proposed for the detection of SARS-CoV-2 to improve the screening capability of suspected contagions. The strengthening of the vaccination campaign represents the most effective means to combat the SARS-CoV-2 infection and prevent severe manifestations of the virus—different classes of vaccines have been developed for this purpose. Further attention on the novel SARS-CoV-2 variant is necessary in order to verify the protection efficacy and virulence reduction of the infective agent in the recent vaccine campaign.

## 1. Introduction

The new coronavirus SARS-CoV-2 (severe acute respiratory syndrome corona virus 2) is responsible for the pandemic Coronavirus Disease 2019 (COVID-19) [1,2,3,4,5,6,7,8,9,10,11,12], an acute respiratory human-to-human transmission disease, which exploded in the last months of 2019, causing more than 49,578,590 cases of infections and about 1,245,717 deaths (ANSA 2020), affecting 192 countries [13,14]. The epicentre is said to be Hubei province in China [15,16,17], the virus originating in November 2019 from bats [18] in close contact with humans [19]. On 12 March 2020, the World Health Organization (WHO) termed the COVID-19 epidemic a pandemic [3,17].

The Johns Hopkins University on 14 March 2021, declared a total of 119,524,282 confirmed cases of COVID-19, including 2,648,320 deaths. The most affected country is the United States (U.S.A.) with 29,399,987 cases and 534,291 deaths, followed by India with 11,359,048 cases and 158,607 deaths, Brazil with 11,439,558 cases and 277,102 deaths, and Russia with 4,331,396 cases and 90,169 deaths. In Europe, the United Kingdom (U.K.) holds the record with 4,267,015 cases and 125,701 deaths, while France is second with 4,105,527 cases and 90,376 deaths. In Italy, a total of 3,201,838 cases and 101,881 deaths was reported, but because of this death rate, it retains the first place in Europe and in the world for the percentage of deaths compared to the total population [1]. COVID-19 clinical manifestations range from asymptomatic, mild, to severe conditions, accompanied by systemic alterations [7,20,21]. The most common significant symptoms are dry cough, shortness of breath, weakness, and fever. Other manifestations include muscle and articular pain, loss of smell and taste, conjunctivitis, cough, fever, and shortness of breath [7,14]. Other reported symptoms are weakness, malaise, respiratory distress, muscle pain, sore throat, loss of taste and/or smell. Severe clinical cases are determined by dyspnoea, hypoxia, respiratory distress, and monoliteral/bilateral interstitial pneumonia [22,23,24].

## 2. Virus Characteristics and Pathogenic Mechanisms

**Figure 1:**
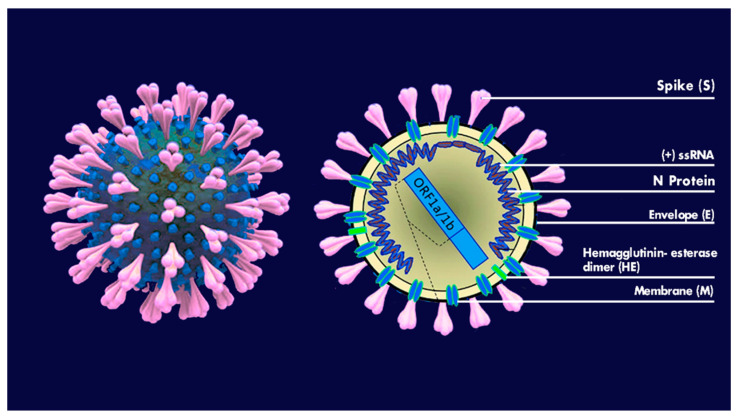
SARS-CoV-2. Spike Glycoprotein. RNA and N Protein, Envelope, Protein M, Hemagglutinin-esterase dimer (HE). Figure designed by Giovanna Dipalma.

Viruses are little microorganisms sized 0.2–0.3 μm up to 1 μm that are able to infect the host cell and bacteria through elective sites and reproduce themselves [6]. The coronaviruses (Coronaviridae) are a virus family of positive-sense-single-stranded ribonucleic acid (RNA) with coat [2], with genome of among 27 and 34 kilobase [3]. The name coronavirus is related to its characteristic look, identified by a bidimensional transmission electron microscope. Coronaviruses have pointed peplomers shaped like a club, which cover their surfaces [19]. They have an extremely elementary structure and some of them have an external cover made of glycoproteins and lipids, taking the name of envelope (E), where inside there exists a protective covering called a capsid, which envelops the viral genome (Figure 1). Four strains of coronaviruses have been noted (α-CoV, β-CoV, γ-CoV, and e δ-CoV), which mainly affect vertebrates. Most studies think that the γ-CoV and δ-CoV interact with birds, but in fact they may also infect mammalians, including humans [23]. The coronaviruses’ genomic structure is characterized by an enveloped RNA genome, with a positive-single strand, which synthesises four main viral structural proteins: spike (S), envelope (E), membrane (M), and nucleocapsid (N) proteins 3–5. The viral envelope contains S, E, and M proteins and the spike protein is responsible for the virus entry mechanism. The S protein has an important role in the bond of the receptor and the access of the virus in the host cells, and therefore is an important element of therapeutic study. The proteins M and E play a fundamental role in the assembly of the virus and the N protein intervenes in the RNA synthesis [20,21,22,23]. The severe acute respiratory syndrome coronavirus (SARS-CoV) and the middle east respiratory syndrome (MERS-CoVs) have a zoonotic origin that seems to always come from bats. With an intermediate host—the SARS-CoVs from civet and the MERS-CoVs from camel and/or dromedary—they are known to cause severe respiratory diseases with frequent deaths as well as persistent fever, dry cough, shivers, stiffening and muscle pain, asthenia, headache, and dyspnoea [6,25,26,27,28,29]. Because of the wide recombination of the genomes of the coronavirus, and high mutability, the SARS-CoV-2 has been identified to have a higher infection rate [30]. It has been highlighted that the natural host of the SARS-CoV-2 is the bat, related to the Rhinolophus, as the genomic sequence of the bat is similar (96.2%) to the RNA and SARS-CoV-2. With respect to the intermediate host, there are studies distinguishing [31] the hypothesis of the pangolin [32,33,34,35]. According to this new version, the first SARS-CoV-2 found in a HU-1 patient in Wuhan, in the first few days of December 2019, was perfectly adapted to the human being. In the months to follow, despite it reproducing trillions of times, substantial and important mutations of genomic transcription, the situation remained unchanged [32]. When the SARS-CoVs originated, it did not immediately infect humans; however, it spread as a result of significant mutations in the first few months of the epidemic via man-to-man transmission. Since the beginning, the SARS-CoV-2 has been a more stable virus than the SARS-CoVs, which also affects the respiratory tract first by encouraging infection [23,36,37]. It has been shown that SARS-CoV-2 is extremely pathogenic, is a powerful suppressor of antiviral immunity, and is an activator of the pro-inflammatory response. The clinical condition of patients with COVID-19 is severe, and this has also been seen in other respiratory viral infections [38,39]. Moreover, the affinity of SARS-CoV-2 for its receptor in the host, ACE2, is 10 to 20 times higher than that of SARS-CoV. Those two features make the SARS-CoV-2 much more infective than the SARS-CoVs [40]. The SARS-CoV-2 virus has a reduced lethality, and high infectivity and resistance, while the SARS-CoV is very lethal, but can be easily extinguished [23]. The bat is surely the primary source of the virus. However, the starting supposition—that the pangolin, a little squamous anteater, was the intermediate host of the SARS-CoV-2 between the bat and the man—has been excluded. In fact, the genomic sequence between the virus of the pangolin and the human is lower (84%) that that of the bat (96%) [30,32]. A second hypothesis is that the virus reached an evaluated step by infecting in an almost asymptomatic way for three months through mutations, some positive, others negative, by increasing the infectivity and reducing the lethality [30]. The SARS-CoV-2 belongs to the genre Betacoronavirus, and it is strictly related to the coronavirus similar to SARS (SARSr-CoV), but it only shares 74.5% of the identity of the genome. Its name is due to the presence on the envelope rich in phospholipids and glycoproteins of spike (S), which are corona-shaped [21,41,42,43,44,45]. Viruses with a lipid coating are generally susceptible to heat and desiccation, and detergent agents. The SARS-CoV-2 virus, thanks to its lipid coating, encourages deactivation with the use of cleaning agents containing alcohol and all cleaning agents that use hot water. However, in SARS-CoV-2, ORF8 and ORF3b proteins, which modulate antiviral and pro-inflammatory responses, are very different from other similar SARS coronavirus, and this confers differences of pathogenesis and transmissibility [38].

## 3. Diagnostics and Screening 

Different diagnostic approaches have been proposed for accurate SARS-CoV-2 detection through serological enzyme-linked immunosorbent assay tests (ELISA), and mucosal buffers analysis [20]. The specimens for SARS-CoV-2 tests are generally obtained from the upper respiratory tract, such as nasopharyngeal/oropharyngeal buffers, saliva, nasal aspirate and wash, or lower respiratory tract. Moreover, specimens from the lower respiratory tract have been proposed to reduce the recurrence of false negative errors [20]. Three main approaches are used for this purpose:

(1) RNA detection test through nucleic acid amplification by RT-PCR procedure.
(2) Antigen tests based on the detection of a specific surface protein viral antigen.
(3) Antibody tests based on the detection of specific antibodies against SARS-CoV-2.

The diagnostic procedures take place through serological tests and salivary, nasal, and oral- pharyngeal buffers. For the serological tests, some detection methods of IgM and IgG are adopted by using a nucleocapsid protein (NP) cross-reactive of another SARSr-CoV Rp3, which is identical to the 2019-nCoV at 92% [20]. An increase in titles of viral antibodies in patients with infection of SARS-CoV-2 has been observed [46,47]. Some studies have focussed on the diagnostic value of the serologic tests for COVID-19 and the development of antibodies against SARS-CoV-2 [46,48]. By trying to detect the presence of antibodies, above all the IgM, quickly produced after the infection, and combined with the real time PCR, the accuracy and sensitivity of the diagnosis is increased [48]. Guo et al. reported the mechanism of antibody response of SARS-CoV-2 in infected patients. By combining antibody tests with qPCR, it is possible to significantly improve the diagnosis of COVID-19 [49,50]. A protocol for the detection of IgM and IgG was determined through the ELISA method, during the antibody response against rNPs purified from the SARS-CoV-2, namely the N proteins that comprise the structure of the helical capsid. A positive detection by PCR was found to be more sensitive compared to the IgM test detected by ELISA assay 5.5 days before the symptoms showed. The efficiency of the ELISA assay was higher compared to the PCR method 5.5 days after the symptoms began. Moreover, the SARS-CoV-2 spike S1 and the nucleocapsid (N) protein serological enzyme linked immunosorbent assay (ELISA) analysis has been proposed with high specificity (99%) for the viral antigen detection [51]. This technique is useful to provide a definite diagnosis and estimation of SARS-CoV-2 prevalence and incidence in a wide population [51]. Moreover, the percentage of positive patients identified is only 51.9% with a single PCR test, but is significantly increased (98.6%) when the ELISA assay is applied for the IgM to PCR negative patients [50,52]. The presence of the N proteins of the capsid has also been significant in other assays, as the antibody response produced against those proteins led to think that there may be immunodominant antigens in the early diagnosis of COVID-19. Therefore, some rapid lateral flow antibody tests (IgM e IgG) were created during the SARS-CoV-2 infection [49]. Moreover, other tests, such as LFIA (lateral flow immunochromatography assay), allow rapid determination of the presence or absence of antibodies towards SARS-CoV-2. They include the SARS-CoV-2 rapid IgG-IgM combined with antibody test kit, which identifies both IgM anti-SARS-CoV-2 and IgM anti-SARS-CoV-2 in whole blood samples, serum, and human plasma in 15 min [53,54,55]. The test was created by Jiangsu Medomics Medical Technologies in China [56] and the working mechanism of the assay is based on the wettability and transport of reactants along the strain of nitrocellulose chromatographically lateral flow [49,52]. When the sample flows along the device, the antibodies anti-SARS-CoV-2 IgM and anti-SARS-CoV-2 IgG, if in the sample, link to the antigens of the SARS-CoV-2, indicated with the colorimetric reagent of colloidal gold [57]. Instead, when the conjugate sample flows along the strain, the antibodies IgM link to the M line, while the antibody IgG links to the G line. If the sample does not contain antibodies against the SARS-CoV-2, there is not signalling of antigen-antibody complex in the test zone and no appearance of a line [57]. Instead, the control line takes colour with the remaining colloidal gold; it links the excess of conjugate by showing that the fluid is correctly migrated along the device. The control area shows up as a red-violet line during the test, to confirm its validity, if they are positive or negative. If the control link does not appear, the test is considered invalid [57]. After several analyses, it has emerged that this test has a sensitivity of 88.66% and specificity of 90.3%. Other tests have also been performed with samples of blood taken by pricking a finger; this kind of test may be used as a test point of care [30,57]. The detection of sensitivity was higher with a test of antibody IgM-IgG combined together, rather than a test made individually to detect IgM or IgG [57]. By the end of April, Abbott had patented their third test for SARS-CoV-2. The test is the serologic test, also called the antibody test. The Abbott test detects the antibody IgG against SARS-CoV-2 [58]. The IgG are produced in the late step of the infection and many of them remain for long time (months or maybe years) after healing. Therefore, this test helps to detect if the patient has been previously infected by SARS-CoV-2 [58]. For this test, it is essential to use dedicated devices: The new test must be used with laboratory tools “*ARCHITECT i1000SR*”and “*i2000SR*” by Abbott, which can conduct up to 100–200 tests per hour. Among the several diagnostic tests developed to detect positivity to the new coronavirus, the antigenic one is the most used for preliminary findings, followed by confirmation with the RT-PCR test. Because of the simplicity of performance without a laboratory analysis, the low time taken for the results, the reliability of results, and the low cost, the United States might make these tests available to perform at home (as pregnancy tests) [58]. False negatives, however, are always a probability, if compared to the standard RT-PCR molecular swab, which is relatively safer. Differences between the molecular and antigenic tests are:

- The molecular test detects the presence of the genome of SARS-CoV-2 with the material contained in the swabs, while the antigenic one detects traces of its antigen (the Spike glycoprotein).
- The antigenic test is performed on a swab, for example oropharyngeal or nasal mucosa, by detecting the presence of the Spike glycoproteins (S) specific to SARS-CoV-2. However, this could result in false negatives, and does not mean that the virus is inactive. Therefore, it is apt to repeat the test a few days later [58].

### 3.1. Molecular Tests

**Figure 2:**
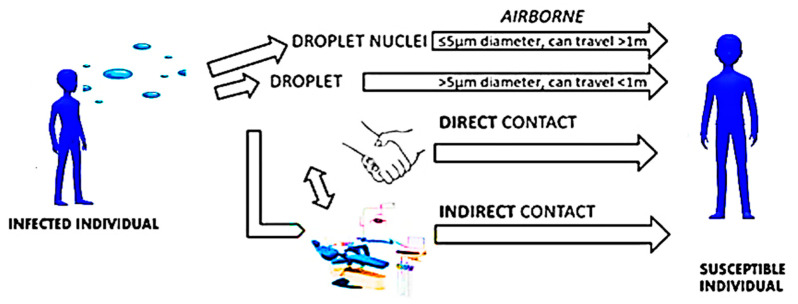
Mechanism of direct and indirect diffusion of the virus Sars-CoV-2 through droplets and aerosol; figure designed by Giovanna Dipalma.

The swab test for the PCR is not immediate but needs a laboratory analysis, which requires between 24 to 72 h, depending on the sampling and conservation system. The antigenic rapid test is a nasal swab performed on the surface where there are specific antibodies that will link to the Spike glycoproteins (S). However, as has been detected in several patients that the new coronavirus mainly proliferates in lung tissues, while it has not been found in the respiratory tract or is in less concentration, it is possible to obtain a false negative [59]. On August 26, Abbott recorded its BinaxNOW with the FDA, an antigenic rapid test also associated with a monitoring app called NAVICA [59]. The test only costs five dollars and gives results in 15 min through the app. It is recorded on a suitable “digital boarding card”, which gives access free in all public places that use the app [59]. This test has been studied in 19 universities, including the University of Rochester Medical Center (URMC), which recently issued a paper of positive data: The results from the 19 sites detected that the test correctly diagnosed coronavirus infection 97.1% times (sensitivity of 97.1%) and correctly gives a negative result of 98.5% (specificity of 98.5%). One advantage of the antigenic test is that does not require sophisticated devices, technical, qualified, or longer times of elaboration [60]. The best method for the clinical diagnosis of COVID-19 is based on the viral insulation of its detection through a sample picked from nasal and pharynx buffer, through buffers picked in other respiratory ways or the sputum—the real time PCR, which is further confirmed by next-generation sequencing [47,60]. This last method is useful, and will continue to determine mature mutations of SARS-CoV-2; however, its use in diagnostics is impracticable [50]. Constant efforts in research and progress in complementary technologies are paving the way for new POC IVD tests (test point of care in vitro). However, the services of IVD tests must be assessed critically before being used for the clinical diagnosis of COVID-19. The IVD tests, namely diagnosis in vitro tests, more used for the diagnosis of confirmation of COVID-19, are based on RT-PCR used at a global level to face the pandemic [49]. A new and solid test, RT-PCR in real time, was developed by Tib-Molbiol, Germany, in collaboration with several partners in the second week of January 2020. It is highly specific for the RNA SARS-CoV-2 and has not cross-reacted with other coronaviruses [60]. The test detects the RNA SARS-CoV-2 by testing the gene RNA polymerase (RdRp) envelope (E) and RNA dependency. The test of gene E has been used for basic screening, while the test of the gene RdRp has been used for confirmation. This kind of assay offers high sensibility and specificity [60]. Another test of wide use is that of the CDC (Center for Disease Control and Prevention). The first panel, including three sets of primer/probe of the gene N, is used both for the generic detection of coronavirus similar to SARS (a set of primer/probe), and the specific detection of SARS-CoV-2 (two sets of primer/probe) [22]. The panel is equipped with a prime/probe, which detects the gene RNase P human (RP) in a sample of the control and in clinical samples [22]. This serologic test detects antibodies produced after SARS-CoV-2 infection. It has a specificity of 99% and sensitivity of 96% and is used to detect SARS-CoV-2 infection in infected patients from 1 to 3 weeks prior. However, the potential cross-reactivity cannot be completely excluded [22]. A few weeks after the first patients were infected, the first sequencies of genome SARS-CoV-2 were detected and recorded in the database of GenBank and GISAID. This led the international scientific community to immediately perform molecular diagnostics. Researchers from the Institute of Virology of Berlin patented some tests to distinguish the SARS-CoV-2 test from SARS-CoV, according to the sequence of the nucleotides of the gene RNA-dependent RNA polymerase (RdRp). The cross-reactivities with other pathogens were validated by using 297 samples picked from patients infected with other common pathogenic respiratory viruses [49]. In another study performed by researchers at the University of Hong Kong, a qualitative rRT-PCR study was developed to detect two different regions ORF1b and N of the genome of SARS-CoV-2. The test gave results within 1 to 15 min. The test of the gene N was used for the screening, while Orf1b was used as test of confirmation. The assay on the region of the gene on ORF1b was about 10 times less sensible than the assay on the gene N [49]. However, this assay is not highly specific for SARS-Cov-2 as the detection of regions ORF1b and N are highly stored also in Sarbecovirus, as well as other strictly linked viruses. The authors have stated that the distinction between SARS-CoV-2 and SARS-CoV may be reached by analysing the sequence of the *“amplicon”* region of DNA or RNA, where mechanisms of amplification and replication originated, which are positive if the results of the RT-PCR are positive [49]. The development of the RT-PCR test with a high specificity for the SARS-CoV-2 genes RdRp/helicase (H) is not sensible for other human coronaviruses and respiratory viruses. Its high sensitivity is effective for low viral load samples and saliva buffers, and plasma and upper respiratory samples [49]. The most important rapid RT-PCR test in real time is Xpert Xpress SARS-CoV-2 of Cepheid, USA [60], which provides results in under 45 min by using the system GenXpert. It is a molecular test point of care (POC), rapid, and automated, which allows to detect SARS-CoV-2 in nasopharyngeal, sputum samples and from nasal washes. The test only requires a minute for preparation of the sample, and uses Cepheid’s cartridge Xpert, acting on more regions of the viral genome. It has obtained Emergency Use Authorization (EUA) by the Food and Drug Administration (FDA) [61]. The most recent revolutionary IVD test is Abbott ID Now COVID-19. On March 27, Abbott received EUA by the US Food and Drug Administration (FDA) for the fastest molecular test point-of-care detection of new coronaviruses (COVID-19). This test provides positive results in just 5 min and negative results in 13 min [61]. It is based on the technique of amplification of the sequency of the specific genome of the viral RNA from SARS-CoV-2. The test may be used anywhere—hospitals, clinics, and medical practices in COVID-19 centres. It is used as a practical touchscreen portable tool (ID Now), and is light (6.6 lbs) and compact [61]. It uses a molecular test for the gene RdRp and uses samples of buffers from throat, nasopharynx, and oropharynx. The kit has 24 tests, positive and negative controls, buffers to pick samples, and pipettes. A preliminary study at New York University raised some doubts about its accuracy, as it may produce false negatives [62,63]. This test is still used and correctly detects several positive cases in a few minutes. It may be necessary to confirm the negative results by integration with a molecular test authorized at high sensitivity [62,63]. Another prospective test is the rapid SARS-CoV-2 by Pharmacyt AG, Germany. It only needs two droplets of blood, giving results within 20 min (Figure 2). Aerosol droplets are considered the main source of transmission of the virus.

**Figure 3:**
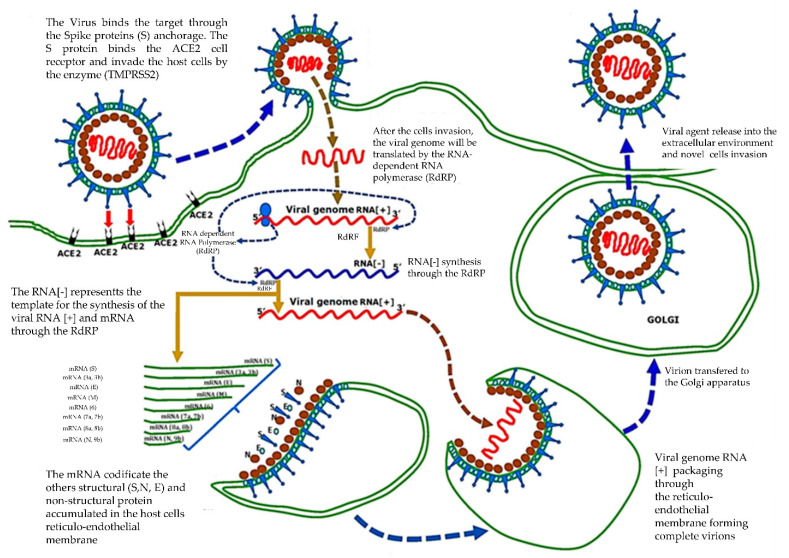
A rapid and sensitive diagnostic procedure for multiple detection of pandemic Coronavirus family members SARS-CoV-2, SARS-CoV, MERS-CoV, and HCoV: A translational research and cooperation between the Phan Chau Trinh Univ. in Vietnam and Univ of Bari “*A. Moro*”. June 2020. ERMPS 2020; 24: 5183–5201, doi:10.26355/eurrev_202006_21713.

**Figure 4:**
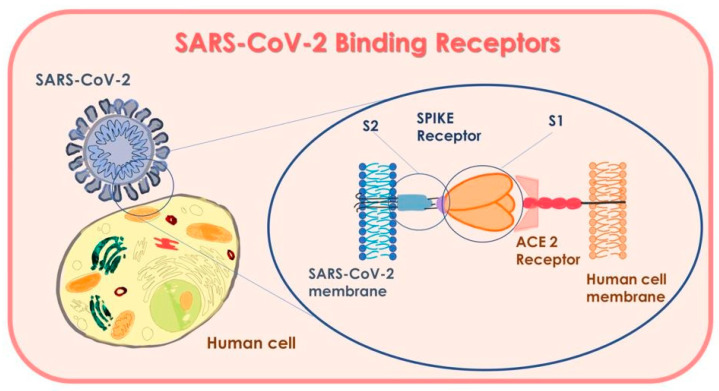
SARS-CoV-2 Binding Receptors.

The results obtained by the rapid test are reflected in those obtained with RT-PCR [64]. Accurate diagnosis of people infected by SARS-CoV-2 is essential to stop the global spread of COVID-19. However, the current diagnostic tests based on RT-PCR are not so reliable [49,62]. Moreover, they can only be performed in well-equipped and highly qualified testing laboratories. Therefore, they have limited usefulness and may not be widely used, for example in developing countries, remote localities, and regions with decentralized laboratories. The delay in diagnosis is contributing to the global spread of COVID-19 [49]. Healthcare workers are required to take precautionary measures to prevent contamination with the virus; for example, dentists, who are the most exposed operators after pulmonologists, infectiologists and intensive care doctors, can perform treatments using lasers in order to reduce aerosol generation [65,66,67,68]. The fast-rapid tests LFIA and CLIA automatized for IgM and IgG may integrate and support the current COVID-19 tests through RT-PCR. However, it is necessary to accurately select the real clinical potentialities of the several existing tests before those are used for COVID-19 diagnosis [69]. Moreover, the detection and molecular investigations may generate some false negatives because of the low viral concentrations in the samples, inadequate quantity and quality of the samples, incorrect transportation and conservation (e.g., long time for transportation), and sample contamination. Evidence from preliminary studies show that COVID-19 asymptomatic patients (subclinical) may show very early but significant changes even before a positive RT-PCR is obtained [49,70]. It has been stated that the most effective strategy to diagnose COVID-19 in suspected patients must include a combination of SARS-CoV-2 RT-PCR with clinical and epidemiological evidence (probability of exposure, signs, symptoms, negative diagnose tests, especially for other respiratory diseases) and findings of chest CT. Samples from respiratory pathways must be taken (at least daily, or every two days) and tested via RT-PCR in patients with negative starting results or high suspicion (or probability) of COVID-19 [49,70,71]. This practice was recently approved by the Food and Drug Administration (FDA) in the United States, concluding that a negative result of a RT-PCR test does not completely exclude SARS-CoV-2 infection and must never be used as a single element for diagnosis. However, SARS-CoV-2 is still evolving; as for the SARS-CoV rate, the current tests showed some limits, with a rate going from 52% to 80% [49]. Currently, the most sensitive diagnostic test in the research of RNA, is a new patent of the University Aldo Moro of Bari and University Pham Chau Trinh, Danang City, Vietnam. On 20 May 2020, a patent in Italy with the number 102020000011701, and the extension numbers EP20197796.4 (Europe), 17/034,407 (USA), PT20202942-FF-P-HK (Hong Kong), was deposited. It was issued on European Review for Medical and Pharmacological Sciences (ERMPS); 2020, June; Doi:10.26355/eurrev_202006_21713. PMID: 32633414 [10]. The whole procedure, including extraction of the RNA and the rRT-PCR, requires less than 3 h and may be performed in any laboratory using PCR [10,71]. Generally, during the acute step, the RNA of the virus SARS-CoV-2 is detectable in respiratory samples. The positive or negative results constitute a response to the infection by SARS-CoV-2, correlated with the clinical and symptomatic condition of the patient. A positive result does not systematically exclude a co-bacterial infection or other viruses belonging to the coronavirus family. In this case, the pathogen agent detected is the cause of the disease [10], thanks to the high sensitivity and specificity degree of the target adopted during the analysis. This diagnosis-kit-system may provide a reliable response to the detection of four strains of coronavirus—SARS-CoV, SARS-CoV-2, H-CoV, and MERS-CoV—and early diagnosis of the infection [10] (Figure 3 and Figure 4).

The buffer test, the “Rapid and sensitive diagnostic procedure for multiple detection of pandemic Coronaviridae family members SARS-CoV-2, SARS-CoV, MERS-CoV and HCoV: a translational research and cooperation between the Phan Chau Trinh University in Vietnam and University of Bari *“Aldo Moro”* [10], is an invention generally referred to diagnosis methods and devices destined for the detection of the virus of the Coronaviridae family. Indeed, in the case of epidemics or pandemics, as the ones caused by the virus of family Coronaviridae, as SARS- CoV-2, SARS-CoV, MERS-CoV, and HCoV, it is important to be able to establish the health status of potential suspected cases in a quick and effective way, not only to save human lives, but also to ensure a more rapid and precise tracking of the spread of infection to adopt necessary health measures. The idea at the base of this invention, together with the rRT-PCR, allows to quantitatively detect the nucleic acid of a plurality of viruses, which represent some diseases linked to the Coronaviridae family, such as by nasopharyngeal and oropharyngeal swabs, sputum, aspirated by the lower and upper respiratory tract, bronchoalveolar washes, and nasopharyngeal aspirates/washes or nasal aspirates, taken from subjects whose doctors have suspected them of having COVID-19 [10].

The adoption of rapid diagnostic tests has different advantages:

1. In the reaction, an enzymatic stabilizer is used, which allows to premix everything and therefore the operator does not need help preparing the mix for the PCR;
2. The primer selected aims at the short fragment and this may allow to reduce the time for the PCR stage to less than 1 h;
3. The cycle of amplification of RNA target of several Coronaviruses occurs in an amplification process, *“One-Step”*, through MPL1 and MPL2. In this single process, both false negatives and positives are highly identified [10].

The rRT-PCR method confirms the full identification of the virus by allowing to exclude the presence of contaminations or false positives (exclusion of viral fragments in the host endothelial cells) or false negatives (exclusion of fragments of virus not detectable as detectable copies). The current scenario of the “pandemic” (COVID-19) requires solid and reliable diagnosis tests to proceed with necessary decisions. A reliable and sensitive test such as this will encourage the organization and definition of countermeasures to face an epidemic and its consequences [10].

#### 3.1.1. Test Sampling

Salivary tests [20,72] tend to confirm the presence of the RNA of the SARS-CoV-2 virus by using specific protocols of real-time PCR [73] for SARS-CoV-2 [55,74,75,76]. The nasopharyngeal and oropharyngeal buffers are invasive: they may cause trauma in the mucosa with slight bleeding and usually induce some reactions such as cough and with a high risk of infection for health professionals [10]. The saliva sample, instead, is easier to perform and tests show positivity to the RNA of SARS-CoV-2. There are three ways for this: through cough, saliva, and salivary glands buffers [77,78]. The saliva taken from the retro pharynx have a higher diagnostic reliability—11 patients out of 12 (91.67%) who did not present with fever and respiratory problems but had suspected symptoms turned out to be positive to the tests. Moreover, 33 patients with negative laboratory tests resulted negative with the salivary test [69,72,77,79]. Even with antibodies against SARS-CoV-2, in saliva samples of the pharynx, the viral RNA was detected in one-third of patients 20 days after and even after a longer period, suggesting that the viral RNA may remain for longer instead of dying with antibodies. A new RNA positive SARS-CoV-2 was detected after two days of negative results in a healed patient; thus, low levels of RNA SARS-CoV-2 may be excreted in saliva even after the symptoms disappear. For this reason, the buffers must be performed for two consecutive days to confirm healing [80] and should be done for at least one week since the appearing of symptoms. More precisely, the infection time of the virus in an apparently healed and asymptomatic patient has not been well defined [80]. Concerning oral buffers, we believe that they are more valid during early diagnosis and at the beginning of the contagion [60]. Indeed, a study on 15 COVID-19 positive patients, by analysing the oral buffers and testing the RNA, found that 8 subjects were positive for the RNA SARS-CoV-2 in oral buffers, four patients (26.7%) had positive nasal buffers, six (40%) had a positive blood test, and three (20%) had positive serum. In a study on 16 patients infected with viral RNA on salivary buffers than nasal buffers, most of the positive results came from oral buffers during the starting step, while more positives came from anal buffers in an advanced step of COVID-19 diagnosis, suggesting that oral buffers may indicate early infection of SARS-CoV-2 but they may not be used as absolute criteria of healing [81]. In a study of saliva picked directly from the salivary glands, taking care not to pollute the buffer from the pharyngeal one, Chen et al. found nucleic acid 2019-nCoV, by stating that salivary glands were infected with SARS-CoV-2 [82]. Thirteen cases were excluded, which resulted positive to the nucleic acid through oropharyngeal buffer among 31 COVID-19 positive patients and four of them (12.90%) were positive in saliva [20,83]. Three of these cases were in critical condition needing respiratory support. This comes from the fact that the nucleic acid SARS-CoV-2 was positive in the saliva secreted by the salivary glands, and this data may be a useful indicator of the gravity of COVID-19 [83,84].

#### 3.1.2. Receptors Mechanism and Interaction

The protein spike on the surface of the new coronavirus has a high affinity with a receptor on human cells. They work as an open-button to the virus: the receptor ACE2, angiotensin converting enzyme 2, object of study for drug therapy, and similar to Coronavirus 19 [85]. The first step of the infection of a virus is that of detecting and recognizing the receptor of the host cell surface to attach, enter, and invade the cell [83]. As the spike of SARS-CoV, SARS-CoV-2 also shares the same receptor as the host cells: ACE2, but with a higher affinity than the spike of SARS CoV [83]. The ACE2 is detected in a significant quantity in type II alveolar cells of the lung epithelial cells in the oesophagus, nose, enterocytes of the ileum and colon, epithelial cells of the gallbladder, myocardial cells, renal tubular cells, and urethral cells of the bladder [24,86,87,88,89,90,91,92]. All those organs with high-expression-cells ACE2 on their cellular walls should be considered as potential organs of easy entry for the virus. Some researchers have shown that the epithelial cells of the oral mucosa are rich in ACE2 receptors [44,49,93]. Moreover, among the several oral sites, some studies have shown that the expression of ACE2 is higher in the tongue than the buccal and gingival tissues and the walls of ducts of the minor salivary glands. These results indicate that the mucosa of the oral cavity may be a potentially high-risk site and easy access of the coronavirus SARS-CoV-2 and result as a Trojan horse, as well as being the first host in the infection of the virus SARS-CoV-2 [84,94]. In turn, the liquid of bronchoalveolar lavage, nasopharyngeal buffers, blood, urine, and saliva coming from patients affected by COVID-19 were RT-PCR positive for SARS-CoV-2 [86,89,90]. In order to assess the ACE2 sites of the oral mucosa, more sites of the same oral cavity were analysed. According to data provided by TCGA (Cancer Genoma Atlas), 32 oral tissues of adjacent sites have been observed: 13 sampled on the tongue, 2 on the base of the tongue, 3 on the wall, 14 from not-well-defined-sites but always in the mouth area. It has been detected that the base of the tongue has a higher expression of receptors of ACE2 than the other sites in the oral cavity [94,95,96].

## 4. Vaccines

Innate immunity, the first stage of defence of our body, plays a fundamental role in protecting ourselves from pathogen agents, by resolving 90% of probable contact infections with bacteria and viruses. The adaptive immunity, more specifically, is potentiated by vaccines. The vaccines’ action is based on a secondary response by exposure of the host subject to the pathogen antigens, inducing the activation of the immune system in the absence of the associated disease [97]. The response after the vaccine administration is comparable to the primary reaction after pathogen exposure but there are reduced effects and related symptoms, creating an immunological memory after initial exposure to the pathogen through adaptive immunity [97]. The intramuscular or intradermic vaccination stimulates the induction of serum IgG but not induction of mucosa IgA [98]. At the moment, all the vaccine candidate for SARS-CoV-2 are administrated intramuscular and very few of them have been projected to induce the immunity of the mucosa. Recent studies confirm that the innate immune system may also be trained [99]. Vaccines increase the basic tone of the innate immunity, as in a training, by increasing the antimicrobic resistance called “agnostic*”*. Myeloid cells, especially macrophages, are determined in the activation, direction, and regulation of the adaptive immune response [99,100]. Lifestyles and chronic tabagism could also represent a factor for heathy immune systems and be summarized as follows: 0-5-30 (0 cigarettes, 5 portions of fruit and vegetables, 30 min of activity) each day. The control of intestinal microbiota is another fundamental element in response of the immune system, associated with the use of probiotics for oral administration [99,100].

### 4.1. Immunity Risk Factors

Obesity influences the immune system and is a risk factor for COVID-19 [78]. The innate immune is also empowered by the vaccine against measles, which protects not only against the virus of measles, but in a higher measure against respiratory infections. The innate immune process of empowerment also explains the fact that babies are less exposed to COVID-19, as most of them are submitted to several vaccines in the first years of life [99,101]. Some recent studies against BCG (tuberculosis) have shown that it activates the threshold of the immune system and may increase resistance to COVID-19 [102,103]. Similarly, it is supposed that the same is valid for the influenza vaccine— it is still to be shown, but is however strongly recommended. The pilot study GWAS (Genome-Wide Association Study), presented in the New England Journal of Medicine in June 2020, in which Humanitas University-IRCCS Humanitas, together with Università Bicocca-Ospedale San Gerardo in Monza and Milan Policlinic participated, has shown that the sensitivity to infect also depends on genetic factors in Italy [99,104]. The study was performed on 1980 Italian and Spanish patients affected by COVID-19 with respiratory failure. The results showed a ‘genic locus ‘in the severe forms of COVID-19, placed on chromosome 3. The gene placed in this region SLC6A20 acts with the protein ACE2 by allowing the virus to access the bond with its Spike protein [104]. A genic cluster 3p21.31 was identified as a locus of genetic susceptibility in patients with COVID-19 with respiratory failure and is related to a probable partnership with ABO blood types. Non-genetic studies have confirmed the involvement of ABO blood types in the exposure to the COVID-19 infection [104]. The genetic data confirm that patients with blood type O have a higher risk of contracting COVID-19 compared to non-O blood types, while blood type A patients have a higher risk compared to the non-A blood type [105]. In the same region, some genes related to chemokines modulators receptors associated with inflammatory responses were detected, which help determine the development and gravity of the COVID-19 disease. These data help us identify at-risk patients [99,105]. As this is a pandemic caused by a new virus and so unknown, it is difficult to foresee the kind of immune response. The strategies used are multiple and so is the dynamics of the vaccines that may lead to protection against the infection [99]. The Coalition for Epidemic Preparedness and Innovations (CEPI), an international organization which encourages the development and stocking of vaccines against microorganisms able to cause new and scary epidemics, is carrying on multiple projects for the realization of vaccines against the SARS-CoV-2 virus [106].

### 4.2. Vaccines Characteristics

Different types of vaccines have been proposed in the literature:

(1) mRNA Vaccine: a sequence of RNA is synthetized in a laboratory and injected into humans. It stimulates the synthesis of the protein, prompting the immune system to produce antibodies that will then act against the virus.
(2) DNA Vaccine: similar to the RNA vaccine—a fragment of DNA is synthetized, which induces cells to produce proteins against which we require an antibody response and subsequent immune response.
(3) Protein Vaccine: in a laboratory, we use sequences of the RNA of the virus to synthetize proteins or fragments of proteins of the viral capsid. Injecting it into the body, together with substances that stimulate an immune response, produces antibodies that prevent respiratory symptoms [99].

The WHO has required the intervention of some world leaders and healthcare partners, including some from the private sector. The biggest investments in history and brains of the most popular scientists have been employed. This has allowed to conclude the several stages of compulsory experimentations, preceded by rigid studies on efficacy and security by reaching the realization of vaccines in time never reached in the past (12 months). There are many pharmaceutical companies (at least ten) that have reached stage 3, a stage in which the vaccine is administrated to a conspicuous number of people in order to assess the degree of immunity produced by it [106] (Table 1 and Table 2). From a total of 230 COVID-19 vaccine candidates obtained after only a year of studies, only a few are currently in use [106]. In July 2020, China was the first country to administer doses of the Sinopharm vaccine (manufactured by the Chinese National Pharmaceutical Group) to the most fragile categories of the population. However, in August, Sputnik V, produced in Russia by the Gamaleja National Center for Epidemiological and Microbiological Research, became the first officially registered coronavirus vaccine [106].

Currently, there are five vaccines to which scientists have issued the conclusive data of stage three, and so are in the condition of been registered and distributed on large scale:

(1) Sponsor: Pfizer, Identification Number: BNT162b2, Development: Germany [107].
(2) Sponsor: Modernatx, Inc; mRNA-1273 Identification N.: NCT04470427 Development: United States. News [108].
(3) Sponsor: University of Oxford; ChAdOx1 nCoV-19 Identification N.: NCT04400838 Development: United Kingdom [109].
(4) Ad26.COV2. S di Janssen Johnson & Johnson Corp.
(5) Sputnik V-Gam-COVID-Vac di Gamaleya Res. Institute. Russia.

The phase 4 trial is an open, non-randomized, parallel group study with historical controls. It is a national cohort study on the efficacy and safety of SARS-CoV-2 vaccines (ENFORCE is an equivalence study that evaluates the efficacy and safety of the new SARS-CoV-2 vaccines approved in the EU). The Pfizer, Moderna, and ChAdOx1 vaccine will be rolled out in Denmark on 10,000 people [110]. In the event of increased availability of vaccines, an additional 2500 people will be included for each vaccine. Subsequent phases can be implemented that include larger sections of the population. Age eligible for study: older than 18 years [110]. The participants will be seen 6 times and followed up for 2 years after the first vaccination, with very detailed follow-up on vaccine efficacy. Safety data will be collected during study visits up to 3 months after the first vaccination [110]. Research and biological material samples will be collected at each visit during the two-year follow-up for a sub-study matched to the main protocol. Starting date, 8 February 2021; estimated primary completion date, 31 December 2024; study completion date, 31 December 2024.

The phase 4 trial will evaluate [110]:

- The minimum protective neutralizing antibody titer (MPNAT); that is the minimum level of antibodies sufficient to protect the patient from infection (at 24 months).
- Number of SARS-CoV-2 positive and confirmed patients (from first vaccination up to 24 months).
- Reports of participants with local and systemic reactions to vaccination (from the first vaccine up to day 90) will be collected.
- Data will be collected from participants who experienced grade 3 and 4 adverse events and any serious adverse events (from the first vaccine up to day 90).

### 4.3. Pfizer Vaccine

**Figure 5:**
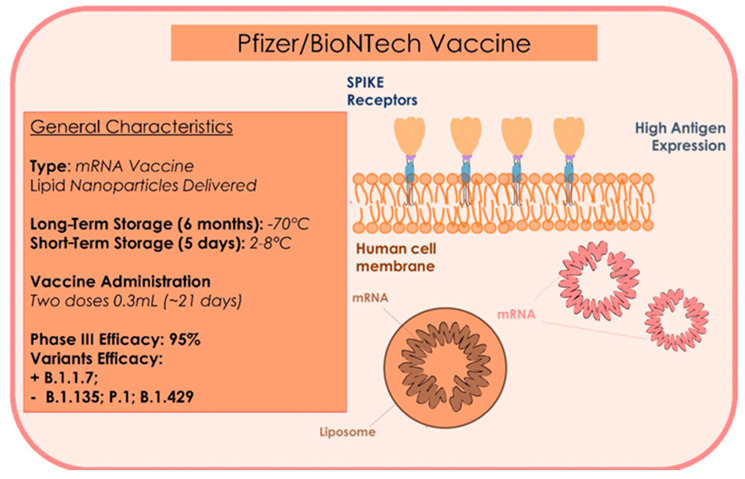
Summary of the type, storage administration, and efficacy characteristics of the Pfizer Vaccine.

This is a messenger RNA vaccine (mRna). Pfizer, together with German company BioNTech has used innovative technology. The vaccine contains lipidic nanoparticles that wrap a strip of genetic material, the messenger Rna (mRna). It uses some fragments of mRNA containing information to produce the glycoprotein Spike (the main antigen of the SARS-CoV-2) by allowing the virus to link to the receptor ACE2 of the target cells (Figure 5). Thus, the infection simulated will encourage the production of specific antibodies, as immunoglobulin G, whose permanence leads to immunity. With this process, people obtain immunization without infecting and being infective [111]. The Pfizer vaccine must be stored at −70 °C for six months. For this reason, recent orientation seeks to make it resistant for at least five days at 4 °C, namely at regular refrigerator temperature [106]. It requires two administrations 21 days apart. In the trial by Pfizer/BioNTech, which has recruited about 44 thousand volunteers, there have been 94 cases of COVID until today: only 10 cases of infection in people who received the two doses of vaccine. On 18 November 2020, the pharmaceutical company Pfizer declared the first stage 3 results on the efficacy of the vaccine BNT162b2 in healthy people. It was a random double blind study, controlled with a placebo [107]. On July 27, the clinical experimentation of stage 3 of the vaccine began. 43,500 people were tested in six countries (United States, Germany, Turkey, South Africa, Brazil, and Argentina). 42% of the global volunteers and 30% of the American volunteers were from different ethnic groups; 41% of all participants and 45% of American participants were aged between 56 and 85 years [107,112]. The data showed that the vaccine was well tolerated. A good part of the side-effects was resolved immediately. No serious or important manifestations were seen; fatigue (3.8%) and headache (2.0%) [106] was reported. From day 21 after administration of the first dose, an efficacy of 95% against COVID-19 was seen. The efficacy in adults aged more than 65 years was higher than 94%. Administration to patients over 16 years is recommended [113]. The side-effects include fatigue, headache, shivers, and muscle pain, primarily after the second dose [113]. As the goal of safety of data required by the American Food and Drug Administration (FDA) for the authorization of use in emergency (EUA) has been reached, the FDA has allowed the registration of patent of vaccine to EUA so that it can be used globally [106]. The United Kingdom was the first country to roll out the Pfizer/BioNTech vaccine on 8 December 2020. The general vaccination strategy plan aims to provide primary cover for elders and people with high comorbidity risk. After two cases of allergic reactions related to the first administration, the agency for the regulation of drugs and healthcare products (MHRA) recommended that patients with a history of immediate anaphylaxis to a vaccine, drug, or food should not receive the Pfizer/BioNTech vaccine. Moreover, in case of a reaction after the first dose, the second dose must not be administrated. The receivers of the vaccine must be monitored for 15 min after the vaccination, with a longer period of observation when indicated after the clinical assessment. A protocol for the management of anaphylaxis and an anaphylaxis package must be available each time the Pfizer/BioNTech vaccine is administrated. Immediate treatment should include 0.5 mg of intramuscular adrenalin (0.5 mL di 1:1000 o 1 mg/mL of adrenalin), with a quick call for help and additional adrenalin IM every 5 min. The healthcare operators who oversee the immunization service must be trained to recognize an anaphylaxis reaction and have familiarity with the technique of resuscitation of a patient with anaphylaxis [113]. There is no sufficient data for a protocol concerning patients with a compromised immune system [113]. The vaccine has shown efficacy of more than 95% in preventing diseases, when tested at least one week after that the participants received a second dose (three weeks after the first one) [106]. The clinical experimentation will continue until 164 confirmed cases of COVID-19 (despite the vaccination) are counted, to obtain further data and assess other parameters of the vaccination. This is done to assesses immunity development in cases verified 14 days after the second dose [111]. The Pfizer-BioNTech vaccine may be less-effective against the B.1.351 variant first identified in South Africa, but offers good protection against the B.1.1.7 English variant [114].

### 4.4. Moderna Vaccine (COVID-19 mRNA-1273 Vaccine)

**Figure 6:**
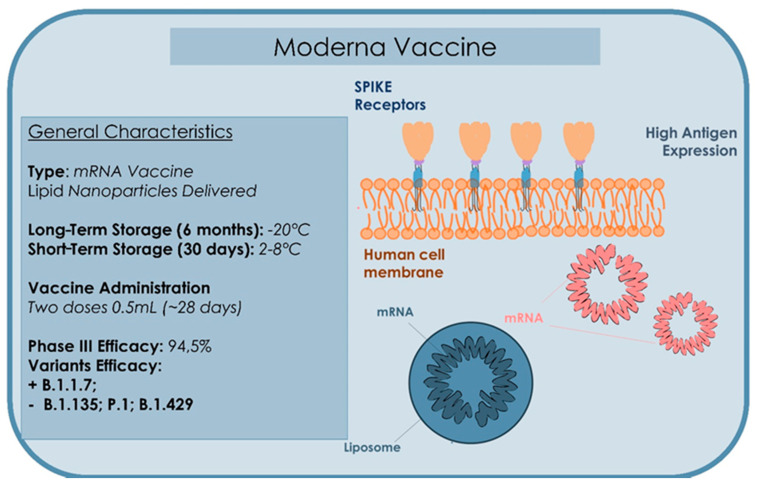
Summary of the type, storage administration, and efficacy characteristics of the Moderna Vaccine.

On 7 December 2020, the FDA approved the large-scale distribution of the Moderna vaccine [83], which showed an efficacy of 94.5%. During the experimentation, 95 cases of the virus were confirmed. The vaccine includes two doses, four weeks apart (Figure 6). It side-effects are fever, muscles pain, and headache. There may be worse effects after the second dose [113]. The most common side-effects (SE) after administration of the two doses are pain in injection site (88.2%), erythema (8.6%), swelling (12.2%), and ipsilateral lymphadenopathy (14.2%). Most of these are of degree 1 (slight) or 2 (moderate); however, a higher incidence of reaction of degree 3 occurred in the group mRNA-1273 and after the second injection. The Data Safety Monitoring Board of the study, appointed by the National Institute of Health of the United States, has observed that 95 cases of COVID-19 were in the placebo group and five in the group vaccine [108]. The study involved more than 30,000 American inhabitants, among which 7000 were aged more than 65 years and 5000 aged younger than 65 years with chronic diseases at high risk. One-third (37%) of the participants was black people, with 6000 Hispanic participants and more than 3000 identified as black [108]. Of the 95 cases, 15 were aged more than 65 years and 20 classified as belonging to several communities (12 Hispanic, 4 black, 3 Asian-American, and 1 multiracial). Moderna has stated that, “the vaccine may be stored in a freezer up to six months and once unfreezed may be stored up to 30 days in a standard refrigerator. This makes the vaccine easier to administrate.” The Moderna vaccine may be less effective against the B.1.351 variant first identified in South Africa, but offers good protection against the B.1.1.7 English variant [114].

### 4.5. Astrazeneca (Oxford)

**Figure 7:**
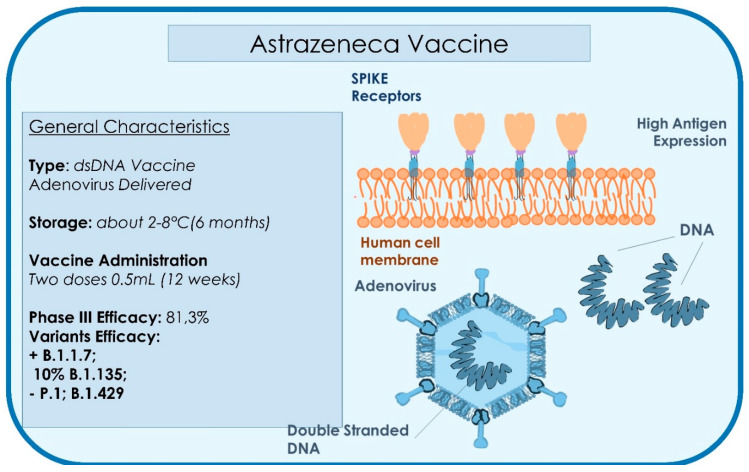
Summary of the type, storage administration, and efficacy characteristics of the AstraZeneca Vaccine.

This vaccine is one of the three approved vaccines (AIFA 30 January 2021), together with the Pfizer and Moderna, reporting 95% efficacy in preventing the disease. The pharmaceutical company used a strain of adenovirus disactivated that infects chimpanzees; it transports a gene containing information to produce the only glycoprotein Spike (S) in the host of the cells, namely the antigen of SARS-CoV-2, which our immune system recognizes by impeding infection (Figure 7) [109,115]. The Astrazeneca vaccine can be administered to patients over 65 years of age, excluding extremely vulnerable subjects or the immunosuppressed. For these categories, the mRNA vaccines is preferred [109,115]. The overall efficacy, initially observed to be 62%, has been increased to 81.3%, as a few thousand volunteers had been wrongly administrated a first dose halved. In these 2700 subjects, of the 23,000 total, the data of efficacy of 90% is close to the 95% announced by its two competitors, Pfizer and Moderna, who have used different technologies [109,116,117,118]. Overall, a total of 131 cases with non-severe infection has been reported, without hospitalizations or deaths. In addition, a wrong dosage led to better results than the planned one [118,119]. The reason for this may be because the reduced dose could have attenuated the response of the immune system to the first contact of the viral vector, by producing lymphocyte T but not the development of antibodies, by subsequently amplifying the reaction at the moment of the second dose after 4–12 weeks [116,119]. Important data highlights that no patient has shown severe virus infections and so it suggests that this vaccine may mitigate the effect of the virus, even when it does not fully immunize it. An important element is the higher ease of conservation and distribution, as the adenovirus vaccine may resist at least six months at standard refrigerator temperature [119]. It is considered that the human lower respiratory tract is protected by the IgG (IgG1 is the most spread), the main kind of antibody in the serum, which is transported in the lung, while the upper respiratory tract is protected by the secretory IgA1 (sIgA1) [98]. It is probable that vaccines will only protect from infection of the lower respiratory tract and would not induce immunity in the upper respiratory tract. This would derive that these vaccines, even if they protect symptomatic diseases, would allow the transmission of the virus, even if in reduced quantity [98]. Therefore, a vaccine to induce sterilizing immunity in the upper respiratory tract as well is desirable. Unfortunately, at the moment, there is none in clinical experimentation [98]. Another important aspect is tolerability, above all in babies, as they usually have a higher reactivity than adults. As many vaccines have important side-effects, it may be necessary to produce low dosage vaccines for this age group, especially for adenovirus (AdV) and mRNA vaccines [98,120,121,122,123,124,125,126,127,128,129,130]. On 30 January 2021, the Italian Agency for Drug Administration (AIFA) confirmed EMA’s assessment of the efficacy (59.5% in reducing symptomatic COVID-19 infections) and benefit/risk ratio favourable of the vaccine and approved the administration of the AstraZeneca vaccine for the prevention of the pandemic, also in consideration of the greater manageability of use of this vaccine. The efficacy of ChAdOx1 nCoV-19 is similar to the efficacy of the vaccine against other lineages [131]. In March 2021, severe thrombosis, an adverse effect, was reported after vaccine administration; further investigation has been planned by the EMA to evaluate product safety [132]. On 12 March 2021, Denmark, Iceland, and Norway suspended the Astrazeneca vaccine campaign due to thrombotic events associated with the drug administration [132]. In Italy, the Italian Agency of Drug (AIFA) banned the use of batch number ABV2856 after three deaths [133]. In Austria, batch number ABV5300 was suspended after the death of a woman. This lot was distributed in 17 countries, including Estonia, Lithuania, Luxembourg, and Latvia, which have also suspended the same batch [124,125].

### 4.6. Janssen Single Dose Vaccine

**Figure 8:**
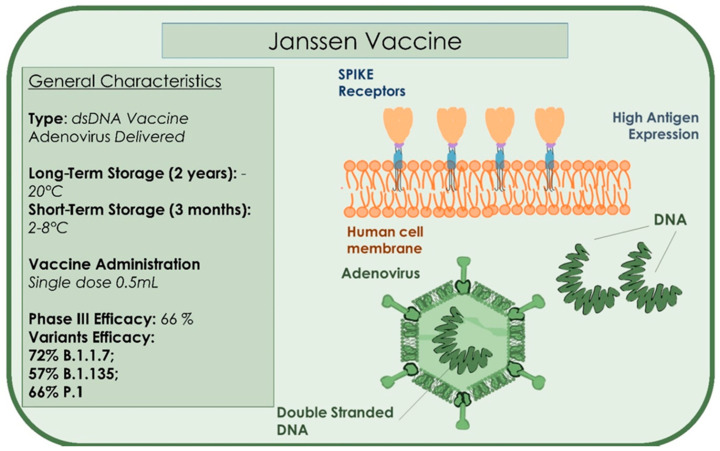
Summary of the type, storage administration, and efficacy characteristics of the Janssen vaccine.

The Janssen vaccine is a single injection COVID-19 vaccine developed by Johnson & Johnson. It is also called Ad.26.COV2S or JNJ-78436725. The vaccine reported 100% efficacy against severe COVID 19 infection, can be administered to people over the age of 18 years, and stored in standard refrigerators (Figure 8) [134]. The Janssen vaccine is easy to store and stable for a maximum of 2 years at −20 °C, and three months if maintained at 2–8 °C [135]. The Janssen vaccine reported complete effectiveness against moderate to severe COVID-19 and 85% against severe forms of the disease. The Janssen vaccine was proven on 43,783 subjects and in 468 symptomatic patients. The vaccine induced different responses on heterogenic viral variants. The vaccine showed an increased effectiveness of 72% in the United States against moderate/severe disease and a lower response of 57% against the South African variant, 501Y.V2 [136]. The Janssen vaccine has also been tested on the Brazilian, British, and South African variants and showed an average effectiveness of 66.9% after 14 days of administration, and 66.1% after 28 days with no safety issues reported [136]. On February 27, the Janssen single-dose vaccine was approved for public administration in the United States and received the approval of the Food and Drug Administration (FDA) [137]. The European Medicines Agency (EMA) is currently evaluating the Janssen vaccine for formal approval and use in Europe on 11 March 2021 [138].

### 4.7. Sputnik V (GAM-COVID-VAC)

**Figure 9:**
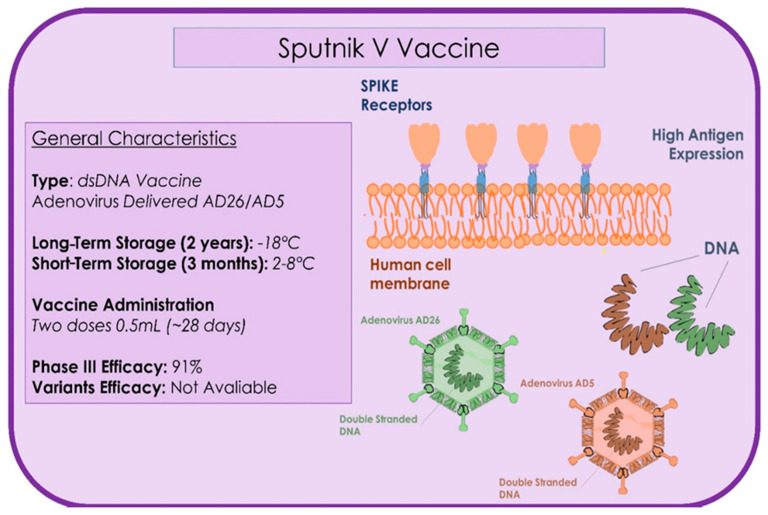
Summary of the type, storage administration, and efficacy characteristics of the Sputnik vaccine.

The Sputnik is a two-doses vaccine with two vectors, rAd26-S and rAd5-S, which express the full-length gene for S. SARS-CoV-2 glycoprotein and is administered at 21 days between the first and second doses (Figure 9) [139]. The Sputnik V recombinant adenovirus (Ad) combined the Oxford-AstraZeneca chimpanzee adenovirus (ChAdOx), the Janssen vaccine (Ad26), and the CanSino vaccine that used Ad5 [136]. On March 4, the EMA launched the rolling review of the COVID-19 Sputnik vaccine [140]. The multicentred study was conducted in 25 healthcare facilities on a total of 21,977 subjects between the test group and the placebo with a 3:1 allocation ratio. The patients presented age >18 years, negative to SARS-CoV-2 [141]. The study effectiveness reported that the antigens induced both cellular and humoral immunity after a single immunization [142]. The Sputnik vaccine’s efficacy was found to be 91.6%. The study reported that the vaccine invoked a high immunity response in healthy subjects with few data on patients over 60 years of age. The vaccine is recommended to be stored at −18 °C, in liquid form and possibility stored at 2–8 °C for global distribution [141]. There is little evidence on novel SARS-CoV-2 strains, but the use of two different viral vectors could represent a potential advantage against the new viral strains [141]. More data are necessary on administration to children, and in pregnant and lactating subjects. The Sputnik has been approved in 16 countries, and represents one of the most effective vaccines (95% effective) [143,144]. The Russian Government has approved the testing of the combination of AstraZeneca-Sputnik V to be more effective against novel SARS-CoV-2 variants [134].

### 4.8. Coronavac/Sinovac

**Figure 10:**
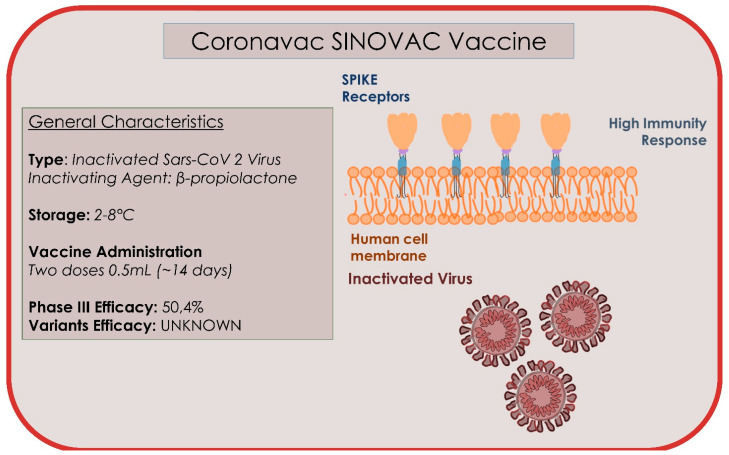
Summary of the type, storage administration, and efficacy characteristics of Coronavac vaccine.

The Coronavac is a traditional vaccine produced by Chinese company Sinovac, Life Sciences (Beijing, China), and contains 3 μg/0.5 mL (equivalent to 600 SU per dose) of inactivated SARS-CoV-2 virus and aluminium hydroxide as an adjuvant. The doses are administered two weeks apart (Figure 10) [145]. The vaccine has been approved in Brazil, China, Indonesia, Cyprus, and Turkey. The inactivated whole virus has several advantages: low production cost, safety, and does not require genetic manipulation. However, it requires specialized laboratory equipment and longer production times than the other methods [146]. Coronavac can be stored at refrigerator temperatures of 2–8 °C, facilitating its use in developing countries. Researchers from the Butantan Institute (Sao Paulo in Brazil) have stated that the Coronavac vaccine would be 78% effective in preventing mild forms of Covid-19 and 100% moderate and severe forms [145]. The trial involved 13,060 adult health workers (11,800 aged 18–59 years) and 1260 elderly participants (60 years and older), who work in units specialized in direct contact with people with possible or confirmed cases of COVID-19; however, the exact data have not been disclosed. The request for approval for emergency use will be submitted to the Brazilian regulatory agency shortly. The director of Butantan Institute, Dimas Tadeau Covas, states that the main goal of the study—to prevent the disease from progressing to moderate and severe forms—has been achieved, despite not being as effective as the mRna vaccines (95%) [145]. The Chinese Coronavac vaccine has shown 78% efficacy in the Brazilian branch of the study, but the exact numbers are not public [145]. The experts reserve the right to provide further data at the moment, which will be communicated in documentation for the request for approval for emergency use that will be submitted to the Brazilian regulatory agency shortly, and in future scientific publication. There would be about 220 confirmed cases of mild disease, 160 in the control group and about 60 in the group that received the vaccine. Based on these data, the effectiveness would be lower than the official 78%. The results of the Coronavac trial in Turkey are different. The effectiveness in preventing mild forms exceeds 90% (26 cases out of 570 volunteers in the placebo group and 3 cases out of 752 vaccinated volunteers), but the population sample is much more modest [145]. The Sinovac vaccine was also included in the Phase 4 study, where a total of 1200 healthy patients aged 18 and over were recruited [145]. On 16 February 2021, uncontrolled open label, all participants will receive two doses of the COVID-19 inactivated adsorbed vaccine (Coronavac) and their data will be followed up for 24 months to analyse safety and immunogenicity data [145]. The total duration of the study is estimated to be 30 months from the start of recruitment (3–4 months). The study will be conducted at the D’Or Institute for Research and Education (IDOR) clinical research centre, located at the Glória D’Or Hospital, in the city of Rio de Janeiro, RJ, Brazil, where the following will be evaluated [145]:

- The number of local and systemic adverse reactions in the first 7 days after vaccination by age group (18–59 years and 60 years or older), attributed to the vaccine.
- Seroconversion rates (two weeks after the second immunization for age group 18–59 years and 60 years or older).
- Frequency of local and systemic adverse reactions up to 28 days after the second dose by age group (18–59 years and 60 years or older), attributed to the vaccine.
- Frequency of serious adverse events up to 12 months after the first dose.
- Cell-mediated immune response (from administration up to 4 weeks after the second vaccination).
- Detection and titration of antibodies against SARS-CoV-2 (from administration, two weeks, 6 months, 12 months, 18 months, and 24 months after the second vaccination).

Confirmed cases of COVID-19, variants of SARS-CoV-2 (VOC), will also be evaluated for up to 24 months after the inclusion.

## 5. Conclusions

The great mutability of the COVID-19 virus, several combinations of factors that play a role in the transmission and evolution of the infection, and multiplicity of the organs involved show that there is still a lot to scientifically explore, as current information is limited and the infection appeared recently with manifestation of an ongoing pandemic without similar cases. The advent of new vaccines offer hope to control the situation. The speed of performance and application on a large scale, however, puts forth big questions on future statistics. Of course, we have changed our social approach and have improved efforts to increase the levels of attention for control of the contagion.

## Figures and Tables

**Table 1:** SARS-CoV2 vaccines phase 3 trials, for each experimentation [106].

Phase 3 Trials SARS-CoV-2 Vaccines			
Manufacturer	Type of Vaccine	General Characteristics	Trial Code
**Sinopharm/China National Biotec Group Co/Wuhan Institute of Biological Product**	*Inactivated Virus*	*SARS-CoV-2 (Vero Cell)*	*CNCTR2000034780*
			*CNCTR2000039090*
			*NCT04510207*
			*NCT04612972*
**Sinopharm/China National Biotec Group Co/Beijing Institute of Biological Product**	*Inactivated Virus*	*SARS-CoV-2 (Vero Cell)*	*NCT04560881*
			*NCT04510207*
**CanSino Biological Inc/Beijing Institute of Biological Product**	*Non-replicating vector*	*Adenovitus type S vector*	*NCT04526990*
			*NCT04540419*
**Garnaleya Research Institute/Russian Health Ministry**	*Non-replicating vector*	*Gam-COVID-Vac Adeno-based (Ad26-RHAd5-S)*	*NCT0453096*
			*NCT0564716*
			*NCT04642339*
			*NCT04656613*
**Janssen Pharmacheutical**	*Non-replicating vector*	*Ad26COV2S*	*NCT04505722ù*
			*NCT04614948*
**Novavax**	*Proteic Sub-unit*	*SARS-CoV-2 rS/Matrix M1-Adjuvant*	*NCT04611802*
			*EUCTR2020-004123-16-GB*
			*NCT045583995*
**Anhui Zhaifei Longcom Biopharmaceutical/Institute of Microbiology Chinese Academy of Sciences**	*Proteic Sub-unit*	*Recombinant SARS-CoV-2 (CHO cell)*	*NCT04646590*
**CureVac AG**	*Rna Vaccine*	*CVnCoV Vaccine*	*NCT04674189*
**Institute of Medical Biology/Chinese Academy of Medical Sciences**	*Inactivated Virus*	*SARS-CoV-2 (Vero Cell)*	*NCT04659239*
**Research Institute for Biological Safety Problem/Kazakhistan**	*Inactivated Virus*	*QarCovid-in*	*NCT04691908*
**Cadila Healthcare Ltd.**	*Dna Vaccine*	*NCov Vaccine*	*CTR/2020/07/026352*
**Bharat Biotech International Ltd.**	*Inactivated Virus*	*SARS-CoV-2 virion*	*NCT04543481*

**Table 2:** SARS-CoV2 vaccines phase 4 trials, for each experimentation [106].

Phase 4 Trials SARS-CoV2 Vaccines			
Manufacturer	Type of Vaccine	General Characteristics	Trial Code
**Pfizer/BioNTech + Fosun Pharma**	*RNA Vaccine*	*BNT162 (3 LNP-mRNAs}*	*NCT04760132*
			*EUCTR2O 21-0000412-28*
			*EUCTR2O 21-0000412-28*
**Sinovac Research and Development Ltd.**	*Inactivated Virus*	*SARS-CoV-2 vaccine*	*NCTO445€595 NCTO4508075*
			*NCTO4502344 NCTO4617483*
			*NCTO465179O*
**Astra Zeneca + University of Oxford**	*Non-replicating vector*	*ChAdOxI-S (AZD1222) (Covishield)*	*ISRCTN89951424 NCTO4516746 NCT04540393 NCT045380S1*
			*EUCTR2020-00 52 26-28-DE*
**Moderna + National institute of Allergy and Infectious Diseases (NIAID)**	*RNA Vaccine*	*mRNA-1273*	*NCT04470427*

## Data Availability

All experimental data to support the findings of this study are available contacting the corresponding author upon request.
